# Population-Specific Associations of Deleterious Rare Variants in Coding Region of *P2RY1–P2RY12* Purinergic Receptor Genes in Large-Vessel Ischemic Stroke Patients

**DOI:** 10.3390/ijms18122678

**Published:** 2017-12-11

**Authors:** Piotr K. Janicki, Ceren Eyileten, Victor Ruiz-Velasco, Khaled Anwar Sedeek, Justyna Pordzik, Anna Czlonkowska, Iwona Kurkowska-Jastrzebska, Shigekazu Sugino, Yuka Imamura-Kawasawa, Dagmara Mirowska-Guzel, Marek Postula

**Affiliations:** 1 Perioperative Genomics Laboratory, Penn State College of Medicine, Hershey, PA 17033, USA; pjanicki@pennstatehealth.psu.edu (P.K.J.); ssugino@hmc.psu.edu (S.S.); 2 Department of Experimental and Clinical Pharmacology, Medical University of Warsaw, Center for Preclinical Research and Technology CEPT, 02-097 Warsaw, Poland; cereneyileten@gmail.com (C.E.); j.pordzik@yahoo.co.uk (J.P.); czlonkow@ipin.edu.pl (A.C.); dmirowska@wum.edu.pl (D.M.-G.); 3 Department of Anesthesiology and Perioperative Medicine, Penn State College of Medicine, Hershey, PA 17033, USA; vruizvelasco@psu.edu (V.R.-V.); ksedeek@pennstatehealth.psu.edu (K.A.S.); 4 2nd Department of Neurology, Institute of Psychiatry and Neurology, 02-957 Warsaw, Poland; ikurkowska@ipin.edu.pl; 5 Genome Sciences Facility, Penn State College of Medicine, Hershey, PA 17033, USA; imamura@hmc.psu.edu

**Keywords:** DNA sequencing, platelets, genetic polymorphism, cerebrovascular stroke, purinergic receptors, large-vessel ischemic stroke, Polish population

## Abstract

The contribution of low-frequency and damaging genetic variants associated with platelet function to ischemic stroke (IS) susceptibility remains unknown. We employed a deep re-sequencing approach in Polish patients in order to investigate the contribution of rare variants (minor allele frequency, MAF < 1%) to the IS genetic susceptibility in this population. The genes selected for re-sequencing consisted of 26 genes coding for proteins associated with the surface membrane of platelets. Targeted pooled re-sequencing (Illumina HiSeq 2500) was performed on genomic DNA of 500 cases (patients with history of clinically proven diagnosis of large-vessel IS) and 500 controls. After quality control and prioritization based on allele frequency and damaging probability, follow-up individual genotyping of deleterious rare variants was performed in patients from the original cohort. Gene-based analyses identified an association between IS and 6 rare functional and damaging variants in the purinergic genes (*P2RY1* and *P2RY12* locus). The predicted properties of the most damaging rare variants in *P2RY1* and *P2RY12* were confirmed by using mouse fibroblast cell cultures transfected with plasmid constructs containing cDNA of mutated variants (FLIPR on FlexStation3). This study identified a putative role for rare variants in *P2RY1* and *P2RY12* genes involved in platelet reactivity on large-vessel IS susceptibility in a Polish population.

## 1. Introduction

The genetics of complex diseases, including ischemic stroke (IS), has been previously investigated in genome wide association studies (GWAS), which identified large numbers of common single nucleotide variants associated with disease susceptibility [1]. While relevant disease pathways have been identified by several GWAS, and one targeted re-sequencing study focused on common genetic variants, IS-associated common variants only explain <10% of variance in disease onset [2]. Therefore, research looking into the missing heritability in IS has been focused on the evaluation of the contribution of low frequency and rare variants [3]. Sequencing studies have revealed that low frequency (i.e., minor allele frequency or MAF between 1% and 5%), and in particular, rare (MAF < 1%) genetic variants, are more likely to have a deleterious effect on health compared to common variants [4,5]. Few re-sequencing studies investigating IS in European populations have been performed [6,7]. These studies showed that low frequency and rare protein coding variants in several genes are associated with stroke (*p* < 1 × 10^−6^) [7].

The pathogenesis of IS is strongly influenced by the activation of platelets and subsequent release of the bioactive materials they transport. Platelets can also exert a far-reaching influence, when activated, by releasing microparticles containing lipids, receptors, proteins, and genetic material into circulation. Apart from their well-established role in hemostasis, platelets have been identified as key players in inflammation, angiogenesis, and central nervous system repair [8]. For that reason, we have selected genes associated with platelet plasma membrane receptors as the target for this re-sequencing study. Only one previous re-sequencing study, in association with IS, was performed in the Polish population, and focused on platelets’ common genetic variants [9].

Thus, we aimed to further investigate the contribution of rare, presumably large effect genetic variants within a selection of previously described 26 genes encoding platelet surface receptors, to IS susceptibility in the Polish population [10].

## 2. Results

**Figure 1:**
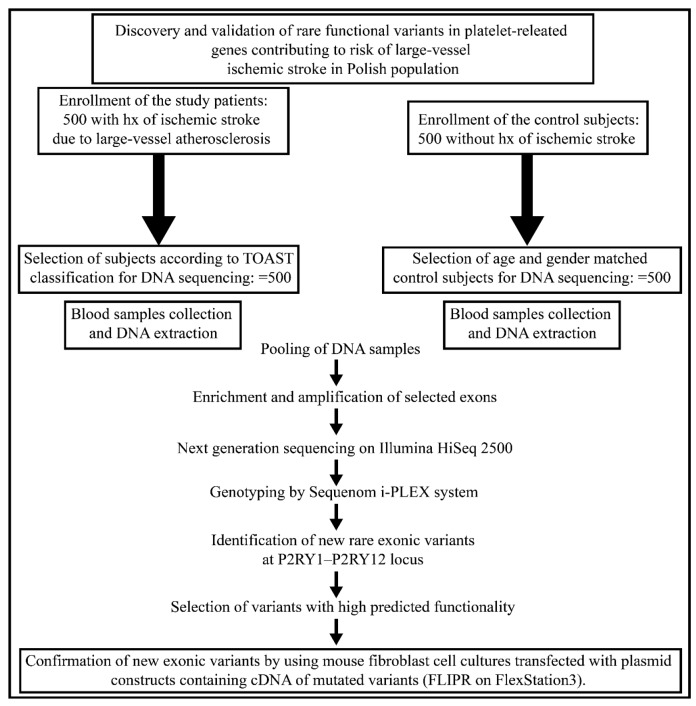
Study-flow diagram. TOAST; Trial of Org 10172 in Acute Stroke Treatment, hx; history.

The study design and flowchart is presented in Figure 1. Pooled targeted enrichment, with the custom Agilent SureSelect capturing kit, resulted in coverage of 99.6%. Sequencing of 10 pools (five each for control and stroke groups) was performed on the Illumina HiSeq2500 sequencer, and generated an average of 36.1 (22.7–45.9 range) million pair-end 101 bp reads, and 5.3 (3–7 range) Gbp per pooled sample consisting of 100 subjects. It corresponds to mean coverage per pool of 12000×, associated with a mean of 120× per individual sample (range 21–369).

The frequency of the investigated damaging allele was presented as combined MAF (cMAF) which encompassed all rare damaging variants in the sequenced gene or region. In total, 477 unique single nucleotide variants (SNVs) with sufficient quality coverage were detected after subsequent stringent quality control. Sixty nine percent (69%) of SNVs were known in the single nucleotide polymorphism (SNP) database (dbSNP) version dbSNP138 (see complete list of known variants in Tables S1 and S2). In all, 248 of the 477 variants (51.9%) were coding variants within target exons of sequenced genes, and the remainder was located in untranslated (introns) and intergenic regions. The final 38 rare and damaging variants were selected out of 129 non-synonymous coding variants based on MAF < 1%, and predicted deleteriousness of single variants, from significant gene-based tests using Combined Annotation Dependent Depletion (CADD), with a minimum scaled CADD score of 10 (corresponding to the top 10% deleterious variants in the genome, as indicated by authors) as a threshold for predicted deleteriousness or damaging properties [11]. The selected variants consisted of 28 known (by dbSNP149 November 2016) and 10 novel (previously not listed) variants.

The rare SNPs (*n* = 38, Table 1) with the most damaging properties were submitted for verification with individual genotyping, of which 31 passed the design of iPLEX Design suite. The individual genotyping was performed in all patients (*n* = 1000) from the original cohort of patients used for pooled targeted re-sequencing. The presence of all 31 variants was verified in at least one carrier from investigated cohorts. In addition, we repeated the calculations in the remaining 605 patients, after exclusion of subjects (from both groups) with a known medical history which could interfere with the burden analysis (including coronary artery disease—CAD, congestive heart failure—CHF, and diabetes mellitus—DM), because the prevalence of these conditions differ between control and stroke groups (Table S3).

The initial statistical analysis was performed using Pearson’s chi-squared test for the comparison of the total (cumulative) frequencies of all cMAF for rare deleterious variants in all sequenced genes between controls and IS cohort, as confirmed by individual genotyping. The obtained data are shown in Table 2. There was a highly statistically significant (*p* = 0.0005) increase in cMAF for all damaging variants in the IS group when compared with controls. Subsequent calculation of cMAF in the study patients remaining after removal of results of subjects with known CAD, CHF, and DM (in both groups) provided a similar difference in the cMAF for all deleterious variants between control and IS cohorts.

The pooled analysis of multiple variants within unique regions or genes, which was based on pooled association test (CMAT), demonstrated a statistically significant difference (*p* = 0.0007) between control and IS cohorts for the *P2RY1–P2RY12* location on chromosome 3 (Table 2). It contained five novel and one known rare and deleterious (CADD score range 12.8–22.8) variant. The region-based, Bonferroni-corrected significance threshold was *p* = 0.0021 (0.05/24 analyzed regions). Similar results were obtained after repeating CMAT analysis for control and IS cohorts remaining after removal of patients with CAD and DM (*p* = 0.03).

**Figure 2:**
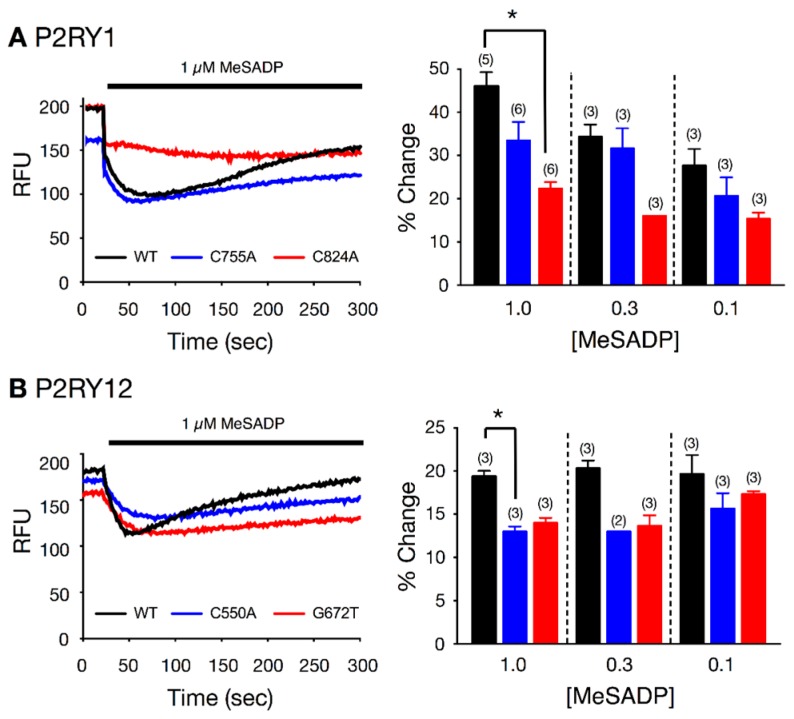
Effect of MeSADP-stimulated fluorescence changes in L cells heterologously expressing *P2RY1* (**A**) and *P2RY12* (**B**) wild type and variant receptors. The left panel (**A**) shows sample fluorescence signals (raw fluorescence units, RFU) from L cells transfected with wild type (black trace), C755A (blue), and C824A (red) *P2RY1* cDNA constructs, before and during MeSADP (1 µM solid line) application. Left panel (**B**) depicts the fluorescence signals (RFU) in cells expressing wild type (black trace), C550A (blue trace) and G672T *P2RY12* receptors. Panels on the right (**A**,**B**) are summary plots showing the mean (+SEM) changes of fluorescence signals following MeSADP application. * *p* < 0.05 employing ANOVA.

To determine whether the SNPs exert a deleterious effect on *P2RY1* and *P2RY12* function, we chose the two presumably most damaging variants (with highest CADD) from each gene, and examined coupling between the heterologously expressed mutant receptors and G protein inwardly-rectifying K^+^ (GIRK) channels in mouse fibroblast (L cells). Figure 2 shows the fluorescence signals of 3 individual wells with L cells expressing wild type *P2RY1* (black trace), *P2RY1* C755A (blue trace), and *P2RY1* C824A (red trace) before and following 2-methylthioadenosine diphosphate (MeSADP) (1 μM) exposure. Following MeSADP application, the fluorescence decreased rapidly, indicative of cell hyperpolarization. It can be seen that the decrease in fluorescence for the C824A variant is approximately half of that obtained with the wild type-expressing L cells. Similarly, the decreased magnitude in fluorescence of C755A *P2RY1*-expressing cells was not as high as that observed in control. The summary plot illustrates that MeSADP produced a dose-dependent increase in hyperpolarization for wild type *P2RY1*-expressing cells, while that obtained with C824A was significantly (*p* < 0.05) attenuated with application of 1 μM MeSADP. On the other hand, the MeSADP-mediated hyperpolarization in C755A-expressing mutants was lower when compared to wild type *P2RY1*, but did not reach significance (*p* = 0.10). The fluorescence tracings shown in Figure 2B depict the effect of MeSADP (1 µM) in L cells expressing wild type *P2RY12* (black trace), *P2RY12* C550A (blue trace), and *P2RY12* G672T (red trace) variants. The summary plot shows that MeSADP (1 µM) exposure produced a significantly (*p* < 0.05) lower change in *P2RY12* C550A-expressing cells than the wild type receptor. The *P2RY12* G672T-expressing cells also exhibited a diminished change (*p* = 0.08) following receptor activation.

## 3. Discussion

In the present sequencing study of a relatively large cohort of 1000 Polish subjects, we investigated the contribution of rare non-synonymous variants in genes coding for platelet plasma membrane surface proteins, to the genetic susceptibility of large-vessel IS. The comparison of MAF for all rare and damaging non-synonymous variants across all sequenced genes and regions demonstrated that there was a statistically significant increase in the cumulative frequency of these variants in the IS group when compared with controls. These results allowed us to examine the contribution of rare coding variants to population variation in IS. By prioritizing rare variants using gene- and region-based tests, we identified novel associations, not previously detected by GWAS. We found association of 6 rare non-synonymous variants in *P2RY1–P2RY12* coding region with large-vessel IS. Grouping rare variants by gene units allowed us to observe associations we were underpowered to detect when only examining single variants, as shown in previous studies of other traits [9]. By doing so, we had not only been gaining insight into the biology underlying platelet membrane proteins, but show that grouping variants by functional annotations could be an effective future strategy. It is important to note that, with exception of one variant, all of the other five investigated rare non-synonymous variants in the *P2RY1–P2RY12* were novel, and never reported before (based on review of >300 rare non-synonymous variants in dbSNP149, and 1000 Genome database listings in March 2017). This might raise the possibility that the observed variants might be specific for the Polish cohort. It is important to mention that Polish patients have been poorly, or not at all, represented in 1000 Genomes and dbSNP. In addition, the recent work of Visschedijk et al. indicates that, at least in case of rare damaging variants associated with ulcerative colitis, the associated variants in the Dutch population could not be replicated in a German replication cohort [12]. Moreover, the first trans-ancestry association study in inflammatory bowel disease performed in several thousands of European individuals and individuals from East Asia, India, or Iran show that the majority of the loci based on MAF > 5% were shared between different ancestry groups [13]. However, this study also found genetic heterogeneity between different ancestry groups for less frequent alleles. Rare variants are even more likely to be specific to a particular population, as was demonstrated by a recent sequencing study in Korean population [14]. It was also demonstrated that the rare variants differ strongly among populations, even between closely related UK populations [15]. As far as the results of our study are concerned, it is interesting that we were able to confirm the presence only one (out of several hundreds) of previously listed rare non-synonymous variants in the *P2RY1–P2RY12* region, which either indicated population-specific distribution of these variants, or might also indicate the limited power of this study. Further studies in both Polish and other populations are obviously needed to confirm that the rare deleterious variants in the purinergic receptor genes region on chromosome 3 are associated with large-vessel stroke, but also with other types of IS (small-vessel and embolic).

Different coding and non-coding SNPs (mostly common type) in *P2RY1* and *P2RY12* were previously evaluated in various IS population, however, the reported results have been conflicting so far [16,17,18,19]. It is worth mentioning that the possible associations of *P2RY12* genetic variants, in a “gain-of-function” haplotype H2 of *P2Y12*, with thromboembolic events (myocardial infarction (MI), IS, or deep venous thromboembolism/pulmonary embolism (DVT/PE)), were not confirmed in a prospective analysis of 14,916 initially healthy American men [17]. The reported conflicting findings could be partly attributable to allelic heterogeneity, case-control selection criteria, phenotype/trait definition, and different population backgrounds. 

The hyperpolarization response observed in the cell culture model served as a measure of adenine diphosphate (ADP)-induced purinergic activation, and was significantly attenuated by the investigated variants. Therefore, it can be assumed that the mutations, when expressed in platelets, could cause subsequent alterations in aggregation and clot formation. The reverse situation (more loss-of-function mutations in the IS group), as observed in this study, indicates that other mechanisms could be responsible for pro-stroke activity associated with P2RY1/P2RY12 receptors. It was previously demonstrated that the ADP-initiated activation of purinergic receptors causes release of endothelium-derived relaxing factor and nitric oxide, with subsequent activation of GIRK channel, hyperpolarization, and vasodilatation of small arteries [20,21]. It can be therefore hypothesized that the attenuation of this mechanism (presence of loss-of-function mutations in P2RY1/P2RY12 receptors) could produce a greater tendency to vasospasm and hypoperfusion associated with IS. It is interesting to note, in this regard, that a protective role for SNVs within coding regions of *P2RY1* in MI was postulated by Ignatovica et al. in the Latvian population [22].

No previous studies addressed the impact or association of rare damaging polymorphisms in *P2RY* genes and IS. On the other hand, different SNPs (mostly common type) in *P2RY1* and *P2RY12* were previously evaluated in various IS cohorts, however, the reported results have been conflicting so far [16,17,18,19,20]. Out of all P2RY receptors, the greatest focus has been put on *P2RY12*, which is expressed in megakaryocyte/platelet lineage, and contributes to progression of thrombosis and hemostasis to cerebrovascular events [23]. To date, most of the studies focused on association between common SNPs within *P2RY12* gene and antiplatelet drug response, mainly clopidogrel [24]. However, only few studies aimed to evaluate the relation between genetic polymorphism within *P2RY* receptor genes’ family, and risk of stroke and data are conflicting. Ziegler et al. found increased risk of ischemic cerebrovascular events in patients with peripheral artery disease treated with clopidogrel, that was associated with of rs6785930 (which is not part of the “H2” haplotype), but not rs6809699 (which is part of the “H2” haplotype) [16]. In the latest study performed in order to investigate the relationship between genetic polymorphisms and poor clinical outcomes in IS patients who underwent stenting for extracranial or intracranial arterial stenosis, four common SNPs within *P2RY12* gene were included (i.e., rs6787801, rs6798347, rs2046934, and rs6801273). Only A-allele carriers of rs2046934 of *P2RY12* had a significant association with an increased risk of clinical outcome events (transient ischemic attack (TIA), IS, MI, and death). In another study, the C allele in *P2RY12* (rs2046934) was predicted to be a protective factor for clopidogrel resistance in IS patients [25]. The latest study aimed to evaluate the association of 2 common SNPs in *P2RY12* (i.e., rs16863323, and rs9859538), 3 common SNPs in *P2RY1* (i.e., rs701265, rs1439010, and rs1371097), and 2 SNPs in *GPIIIa* gene, as well as their interactions with antiplatelet drug responsiveness, and adverse clinical events after minor IS. The high-risk interactive genotypes (i.e., rs16863323TT in *P2RY12*) were independently associated with poor antiplatelet drug responsiveness and increased risk of primary outcomes, defined as composite adverse events of recurrent IS, MI, and death within 90 days after treatment [26]. However, in a prospective analysis of 14,916 American men, there was no evidence for an association of any of the variants or the haplotype H2 (composed of dbSNP rs10935838, rs2046934, rs5853517, and rs6809699) tested with risk of MI, or IS [17].

It has been previously reported that genetic variability of P2RY2 receptor had substantial influence on occurrence of IS. In 237 Japanese patients with a history of IS, five single SNPs within *P2RY2* gene (rs4944831, rs1783596, rs4944832, rs4382936, rs10898909) were genotyped, and using a dominant model for rs4944832 phenotype, it was found that the GG genotype of this SNP is a genetic marker for IS, particularly in women. Moreover, the overall distribution of the haplotype defined by rs1783596–rs4382936–rs10898909 was significantly different between IS and the control groups [27]. The reported conflicting findings could be partly attributable to allelic heterogeneity, case-control selection criteria, phenotype/trait definition, and different population backgrounds.

Our confirmatory in vitro results also indicate that the MeSADP-mediated hyperpolarization of L cells expressing *P2RY1* and *P2RY12* variants was attenuated when compared to cells expressing the wild type receptors. These findings suggest that the signaling is likely altered in platelets expressing the mutant P2RY receptors.

## 4. Materials and Methods

### 4.1. Patients

The local ethics committee of the Institute of Psychiatry and Neurology, Warsaw, Poland, approved both the study protocol (including DNA sample collection and genotyping) and the informed consent form (identification code KB IPiN 06/2011). The study was conducted in accordance with the current version of the Declaration of Helsinki at the time when the study was designed, and informed written consent was obtained from all enrolled patients. The study population consisted of 500 patients with the diagnosis of the acute IS based on clinical features according to the World Health Organization definition and always supported by brain imaging (CT or MRI) that was selected for the existing dataset, as previously described [9,28,29,30]. Briefly, it included information about patients’ demographics, comorbidities, laboratory findings, and the course of stroke. Based on the Trial of Org 10172 in Acute Stroke Treatment (TOAST) classification, we included (i) all patients classified as having IS due to large-vessel atherosclerosis, and (ii) a subset of patients classified as having IS of unknown etiology, provided they had at least 50% stenosis of the carotid artery ipsilateral to the infarct side, and no evidence or no history of atrial fibrillation. The controls consisted of 500 age- and gender-matched patients, free of stroke, with multiple risk factors for cardiovascular disease or present CAD. Blood sampling and DNA extraction was performed as described before [9]. 

### 4.2. Genotyping

The target for genotyping consisted of 26 genes (Table 3) coding for platelet plasma membrane functions and containing total of 241 exons, as well as 10 flanking bases beyond each exon on both sides, which were selected using the human (*Homo sapiens*, hg19, GRCh37, February 2009) database. Pooled targeted enrichment of DNA, from 500 Polish large-vessel IS patients (100 individuals per pool × 5 pools) and 500 age-, gender-matched control patients (without any type of stroke history) (100 individuals per pool × 5 pools), was performed using a custom-made kit (SureSelect from Agilent Santa Clara, CA, USA) in accordance with the manufacturer’s instructions. The customized library was created by the SureDesign platform from Agilent Technologies, with average coverage of 99.6% of the selected exons. A detailed description of sequencing and data analysis is given in the Materials and Methods Supplementary Materials.

#### 4.2.1. Statistical Tests and Calculations

The initial analysis was performed by Pearson’s chi-squared test to ascertain statistical significance for the difference in the total number of alleles in re-sequenced genes containing damaging, rare, non-synonymous variants between controls and patients with large-vessel ischemia. The frequency of the investigated damaging allele was presented as cMAF, which encompassed all rare damaging variants in the sequenced gene or region. Since all observed genes were selected for MAF < 0.05, gene- or region-based burden analysis with the cumulative minor-allele test (CMAT) test (10,000× permutations) was performed in all targeted regions separately, to estimate the statistical significance of the observed differences in the accumulation of non-synonymous SNVs in large-vessel stroke in the investigated cohort when compared to controls. Statistical significance threshold was adjusted to number of target regions re-sequenced in the study (Bonferroni correction).

#### 4.2.2. Sample Size and Power Considerations

In power calculations, instead of using individual rare variants, we decided to use predicted cMAF for all deleterious rare variants in single genes or regions. In this study, we have followed a self-sufficient, closed-form, maximum-likelihood estimator for allele frequencies that account for errors associated with sequencing, and a likelihood-ratio test statistic that provides a simple means for evaluating the null hypothesis of monomorphism [31,32]. Unbiased estimates of allele frequencies 10/*n* (where *n* is the number of individuals sampled) appear to be achievable, and near-certain identification of a SNP requires a cMAF (which is 10 variants for all pooled samples in the given cohort cMAF ~ 0.01). In addition, because the power to detect significant allele-frequency differences between two populations is limited, we set both the number of sampled individuals (500 in the cohort) and depth of sequencing coverage in excess of 100. The level of significance (*p* = 0.001) was assumed for a Fisher’s exact test for frequency differences in cMAF between populations.

### 4.3. Fluorescence-Based Functional Assay for P2RY1 and P2RY12 Receptor Activation in L Cells

Mouse fibroblast (L cells) cultures were transiently transfected by electroporation with cDNA constructs of the identified purinergic receptor genes within the pcDNA3.1 plasmid vector. GIRK channels, effectors for purinergic receptors, are not natively expressed in L cells. Thus, L cells were co-transfected with the *GIRK*4 S143T cDNA construct. The heterologously expressed purinergic receptors were stimulated with the *P2Y1* and *P2Y12* selective agonist, MeSADP. Purinergic receptor stimulation in this system cause specific G proteins to activate GIRK channels, resulting in membrane hyperpolarization (details provided in the Materials and Methods Supplementary Materials).

## 5. Limitations

The main limitation of the study is the lack of verification of observed variants and its association with IS in the independent verification cohort. It should be, however, noted that several previous studies demonstrated that the rare variant associations, because of their private character, are often limited to very limited populations, and are very hard to reproduce, unless the verification groups are very large (in this case, several tens of thousands patients). The other limitation of the study is that only a fraction of all known relevant genes related to platelet reactivity were sequenced in this study. Since the aim of our study was to analyze the impact of rare variants within genes related to platelet reactivity on IS risk, we did not measure platelet reactivity and its association with genetic polymorphism. Also, important limitation of the study was that we matched controls based on gender and age; however, comorbid clinical factors such as DM, CAD, or heart failure were observed more often in the control group. Based on the results of the multivariate logistic regression, after correcting for these clinical factors, one can conclude that the impact of observed polymorphism is unrelated to well-known risk factors for atherothrombotic disease, which further increases the strength of the results. Finally, the iPLEX-Sequenom method used with individual genotyping has a very high level of specificity (~100%), and its sensitivity depends very much on the primer design and stringency, which is usually optimized for detection of frequent SNPs, but not so much for singletons (e.g., only one mutated allele among hundreds of wild type alleles). We did our own spot check to confirm the heterozygous calls, and they were made with high confidence. However, because neither original Sequenom’s calling algorithm nor our post-processing scripts have been optimized for detection of very rare variants, we may have missed other samples with heterozygous or alternate allele homozygous genotypes.

## 6. Conclusions

In summary, the results of our study indicate that the distributions of investigated rare deleterious variants in the coding regions of selected platelet membrane genes (and in particular, located in the region of purinergic receptor P2RY1–P2RY12), in the cohort of Polish patients, could be associated with the large-vessel IS. The mechanisms by which these variants interact with the purinergic transmission and IS remains unclear, and will require further investigations. It is also not certain if our findings could be translated directly to other populations, as the variants driving the observed associations are rare, and appear to be limited to the Polish population. Further studies in larger, and also different populations, would be needed to clarify this question.

## Figures and Tables

**Table 1:** List of all rare (predicted MAF < 0.1%) non-synonymous and deleterious single nucleotide variants observed in the investigated Polish patients (*n* = 1000) after pooled resequencing of exons in 26 genes.

Chr	Gene	Position	Ref	Alt	dbSNP149	cDNA	Protein AA	CADD	MAF Ctrl	MAF Stroke
chr3	*ITPR1*	4714920	A	G	rs35789999	c.A2260G	p.M754V	15.92	0.0014	
chr3	*ITPR1*	4716885	C	T	rs201519806	c.C2687T	p.A896V	12.80		0.0011
chr3	*ITPR1*	4774887	G	T		c.G5147T	p.G1716V	15.77		0.0014
chr3	*ITPR1*	4821291	G	T	rs373973399	c.G6160T	p.A2054S	20.2		0.0013
chr3	*ITPR1*	4842276	G	A	rs201144431	c.G6910A	p.A2304T	16.91	0.0015	0.001
chr17	*GP1BA*	4837662	T	C	rs201408072	c.T1763C	p.V588A	15.12	0.0013	
chr3	*RAF1*	12641707	C	T	rs555034652	c.G934A	p.V312M	12.39		0.0017
chr1	*PTAFR*	28477192	T	C	rs138629813	c.A341G	p.N114S	20.8		0.0018
chr1	*PTAFR*	28477408	C	A		c.G125T	p.R42L	14.45	0.0015	
chr17	*ITGA2B*	42453084	C	T	rs74988902	c.G2602A	p.V868M	13.50		0.0014
chr17	*ITGA2B*	42455791	G	A	rs200481952	c.C2033T	p.A678V	20.4		0.0015
chr17	*ITGA2B*	42457474	G	A	rs548977341	c.C1648T	p.R550W	22.3	0.0018	
chr17	*ITGA2B*	42463054	G	C	rs76066357	c.C439G	p.L147V	11.09		0.0025
chr17	*ITGB3*	45363765	A	G	rs56173532	c.A754G	p.I252V	13.67	0.0012	
chr17	*ITGB3*	45376796	G	A	rs144884023	c.G1813A	p.G605S	35		0.0013
chr19	*PTGIR*	47126849	G	A	rs4987262	c.C634T	p.R212C	22.3		0.0034
chr5	*ITGA2*	52344487	A	G	rs55973669	c.A517G	p.I173V	12.10		0.0022
chr19	*GP6*	55543660	G	A	rs199588110	c.C172T	p.R58C	18.28	0.0025	0.0016
chr19	*GP6*	55543660	G	A	rs199588110	c.C172T	p.R58C	18.28	0.0023	
chr19	*GP6*	55543692	C	T	rs750889036	c.G140A	p.R47Q	10.75		0.0019
chr11	*FERMT3*	63974970	T	G	rs759179590	c.T134G	p.V45G	33	0.0035	0.0042
chr11	*FERMT3*	63974995	C	G	rs142815441	c.C159G	p.I53M	12.17	0.0013	
chr11	*FERMT3*	63978538	G	A	rs762181713	c.G409A	p.E137K	29.0		0.0014
chr11	*P2RY2*	72945434	T	C	rs148391446	c.T230C	p.V77A	14.97		0.0017
chr11	*P2RY2*	72945799	A	G	rs141776297	c.A595G	p.S199G	15.02		0.0021
chr11	*P2RY2*	72945799	A	G	rs141776297	c.A595G	p.S199G	15.02	0.0019	0.0026
chr11	*P2RY2*	72946279	T	C	rs74472890	c.T1075C	p.S359P	12.54	0.0024	
chr3	*GP9*	128781048	G	A	rs3796130	c.G466A	p.A156T	12.21	0.0015	
chr3	P2RY12 *	151055962	C	A		c.G672T	p.R224S	15		0.0021
chr3	P2RY12 *	151056084	G	T		c.C550A	p.L184I	15.13		0.0013
chr3	P2RY1 *	152554155	G	A		c.G584A	p.R195H	18.35	0.0012	0.0034
chr3	P2RY1 *	152554326	C	A		c.C755A	p.S252Y	22.4		0.0015
chr3	P2RY1 *	152554395	C	A		c.C824A	p.P275H	22.8		0.0013
chr3	P2RY1 *	152554482	C	T	rs868057570	c.C911T	p.A304V	12.84		0.0026
chr1	*PEAR1*	156878116	C	T		c.C1099T	p.R367W	18.01		0.0012
chr1	*SELP*	169576246	G	A		c.C1460T	p.A487V	12.76	0.0017	
chr1	*SELP*	169581608	G	A	rs139249907	c.C808T	p.R270X	13.86		0.0016
chr3	*GP5*	194117640	C	A		c.G1372T	p.A458S	10.81		0.0012

* Functional and damaging variants selected for FlexStation3 analyzes.

**Table 2:** List of cumulative minor allele frequencies (cMAF) for damaging non-synonymous variants in the individually genotyped subjects from the control (ctrl) and study (stroke) groups in all patients used for pooled sequencing (left panel) and remaining patients after subtracting cardiac conditions (right panel).

Gene	All Individuals (*n* = 1000). Number of Variant Carriers for Each Locus and Cohort in Brackets			Subjects without Cardiac Disease (*n* = 605). Number of Variants Carriers for Each Locus and Cohort in Brackets		
Region	cMAF ctrl	cMAF stroke	CMAT P/Fisher	cMAF ctrl	cMAF stroke	CMAT P/Fisher
*GP6*	0.006 (3)	0.002 (1)	0.3700	0.000 (0)	0.000 (0)	NA
*ITGA2*	0.000 (0)	0.004 (2)	0.4900	0.0000 (0)	0.002 (2)	0.51
*ITGA2B/ITGB3*	0.004 (2)	0.014 (7)	0.1200	0.005 (2)	0.006 (5)	0.62
*ITPR1*	0.004 (2)	0.006 (3)	0.4900	0.0025 (1)	0.002 (2)	0.90
*P2RY1/P2RY12*	0.002 (1)	0.02 (10)	0.0007 **	0.0025 (1)	0.123 (10)	0.002 **
*P2RY2*	0.004 (2)	0.006 (3)	0.2100	0.0006 (1)	0.004 (3)	0.62
*PEAR1*	0.0000 (0)	0.002 (1)	1.0000	0.000 (0)	0.000 (0)	NA
*PTAFR*	0.0000 (0)	0.002 (1)	1.0000	0.0000 (0)	0.001 (1)	0.99
*SELP*	0.0000 (0)	0.004 (2)	0.1200	0.0000 (0)	0.002 (2)	0.14
*PTGIR*	0.0000 (0)	0.004 (2)	0.2500	0.0000 (0)	0.002 (2)	0.26
Total ^#^	0.02 (10)	0.064 (32)	0.0005 *	0.0125 (5)	0.033 (27)	0.03 *
OR			3.4 (7.6–6.9)			2.8 (1.1–7.3)

** Statistical significance (*p* value) calculated using burden CMAT test (for data with cMAF for variants in present both control and stroke groups) or Fisher exact test (for variants with cMAF only in one studies group and not observed in another group). * Statistical analysis performed using Pearson’s chi-squared test; ^#^—combined cMAF for all observed damaging variants in one of the study group. OR—odds ratio.

**Table 3:** List of sequenced genes involved with the platelet membrane functions.

Gene	Protein Product	Chr Location
*P2RY2*	purinergic receptor P2Y, G-protein coupled, 2	11q13.5-q14.1
*P2RY12*	purinergic receptor P2Y, G-protein coupled, 12	3q25.1
*P2RY1*	Purinergic receptor P2Y, G-protein coupled, 1	3q25.2
*ITGB3*	integrin, beta 3 (platelet glycoprotein IIIa, antigen CD61)	17q21.32
*ITGA2B*	integrin, alpha 2b (platelet glycoprotein IIb of IIb/IIIa complex, antigen CD41)	17q21.32
*GP5*	glycoprotein V	3q29
*GP9*	glycoprotein IX	3q21.3
*GP6*	glycoprotein VI	19q13.42
*GP1Bα*	glycoprotein 1bα	17p13.2
*GP1BB*	glycoprotein Ib (platelet), beta polypeptide, antigen CD42c	22q11.21
*ITGA2 (GPIa)*	integrin, alpha 2 (alpha 2 subunit of VLA-2 receptor, antigen CD49B)	5q11.2
*ADRA2A*	adrenoceptor alpha 2A	10q25.2
*TBXA2R*	thromboxane A2 receptor	19p13.3
*HTR2A*	5-hydroxytryptamine (serotonin) receptor 2A, G protein-coupled	13q14-q21
*F2R (PAR-1)*	proteinase-activated receptor 1 (PAR1), coagulation factor II (thrombin) receptor	5q13
*F2RL3 (PAR-4)*	protease activated receptor 4 (PAR-4), coagulation factor II (thrombin) receptor-like 3	19p12
*PEAR1*	platelet endothelial aggregation receptor-1	1q23.1
*GNB3*	guanine nucleotide binding protein (G protein), beta polypeptide 3	12p13
*CD148*	receptor-type protein tyrosine phosphatase	11p11.1
*ITPR1*	inositol 1,4,5-trisphosphate receptor, type 1	3p26.1
*CD36*	thrombospondin receptor, antigen CD36	7q11.2
*CD40*	TNF receptor superfamily member 5, antigen CD40	20q12-q13.2
*EPR1*	effector cell peptidase receptor 1	17q25
*PECAM-1*	platelet/endothelial cell adhesion molecule 1	17q23.3
*FERMT3*	ferritin family member 3	11q13.1
*PTAFR*	platelet-activating factor receptor	1p35-p34.3
*PTGIR*	prostaglandin I2 (prostacyclin) receptor (IP)	19q13.

## Supplementary Materials

Supplementary materials can be found at www.mdpi.com/1422-0067/18/12/2678/s1.

## Abbreviations

IS	Ischemic stroke
GWAS	Genome wide association studies
MAF	Minor allele frequency
SNVs	Single nucleotide variants
SNPs	Single nucleotide polymorphism
CADD	Combined Annotation Dependent Depletion
TOAST	Trial of Org 10172 in Acute Stroke Treatment
CAD	Coronary artery disease
CHF	Congestive heart failure
DM	Diabetes mellitus
cMAF	Combined MAF
CMAT	Cumulative minor-allele test
SNP	Single nucleotide polymorphism
GIRK	G protein inwardly-rectifying K^+^
MeSAD	2-Methylthioadenosine diphosphate
TIA	Transient ischemic attack
