# Supplementary material for: Population-Specific Associations of Deleterious Rare Variants in Coding Region of P2RY1–P2RY12 Purinergic Receptor Genes in Large-Vessel Ischemic Stroke Patients
Source: Int J Mol Sci. 2017 Dec 11;18(12):2678. doi: 10.3390/ijms18122678 (PMC5751280; doi:10.3390/ijms18122678)
Supplement: Supplementary file 1 [file ijms-18-02678-s001.pdf]

Population-Specific Associations of Deleterious Rare Variants in Coding Region of *P2RY1–P2RY12* Purinergic Receptor Genes in Large-Vessel Ischemic Stroke Patients

Piotr K. Janicki [1], Ceren Eyileten [2], Victor Ruiz-Velasco [3], Khaled Anwar Sedeek [3], Justyna Pordzik [2], Anna Czlonkowska [2,4], Iwona Kurkowska-Jastrzebska [4], Shigekazu Sugino [1], Yuka Imamura-Kawasawa [5], Dagmara Mirowska-Guzel [2] and Marek Postula [1,2,*]

[1] Perioperative Genomics Laboratory, Penn State College of Medicine, Hershey, 17033 PA, USA; pjanicki@pennstatehealth.psu.edu (P.K.J.); ssugino@hmc.psu.edu (S.S.)
[2] Department of Experimental and Clinical Pharmacology, Medical University of Warsaw, Center for Preclinical Research and Technology CEPT, 02-097 Warsaw, Poland; cereneyileten@gmail.com (C.E.); j.pordzik@yahoo.co.uk (J.P.); czlonkow@ipin.edu.pl (A.C.); dmirowska@wum.edu.pl (D.M.-G.)
[3] Department of Anesthesiology and Perioperative Medicine, Penn State College of Medicine, Hershey, 17033 PA, USA; vruizvelasco@psu.edu (V.R.-V.); ksedeek@pennstatehealth.psu.edu (K.A.S.)
[4] 2nd Department of Neurology, Institute of Psychiatry and Neurology, 02-957 Warsaw, Poland; ikurkowska@ipin.edu.pl
[5] Genome Sciences Facility, Penn State College of Medicine, Hershey, 17033 PA, USA; imamura@hmc.psu.edu
* Correspondence: mpostula@wum.edu.pl; Tel.: +48-221-166-160

SUPPLEMENTAL MATERIALS

Materials and Methods Supplement.

1. Sequencing and analysis

The enriched libraries were sequenced using 101 bp paired-end mode on an Illumina HiSeq 2500 sequencer. Sequenced samples (consisting of FASTQ files) were aligned using the BWA-MEM aligner (BaseSpace Labs, Illumina, https://basespace.illumina.com) to a human reference genome version 19, using following tools: BWA version 0.7.13 (https://github.com/lh3/bwa), SAMtools version 1.3 (https://github.com/samtools/samtools), Picard version 2.1.1 (https://github.com/broadinstitute/picard). Aligned reads were recalibrated, sorted, marked for read duplication, and realigned near insertion/deletion (indels) using Genome Analysis ToolKit (GATK 3.7-0, http://www.broadinstitute.otg.gatk). Subsequent variant calling and filtrations were performed using generated from bam files (without removal of duplicate reads, as recommended for pooled sequencing) using Galaxy platform (https://usegalaxy.org). Firstly, bam files were sliced using the Samtools (Galaxy version 2) by the genomic regions containing exonic targets. Secondly, the genetic variants in the genomic targets were annotated using MPileup call variants (Galaxy Version 2.1.1)

routine. The resulted pileup files were processed by VarScan2 software (Galaxy version version 0.1) for the final detection of variants. After final reads assembly by VarScan software and passing quality filters the subsequent screening of sequenced exons (in vcf format) was performed against dbSNP138 using wAnnovar software [14, 15]. The output files (one for each pool) from previous step was annotated by the dbNSFP version 3.2a software (https://sites.google.com/site/jpopgen/dbNSFP). The dbNSFP is an integrated database of functional annotations from multiple sources for the comprehensive collection of human non-synonymous single variants polymorphisms (nsSNVs). Its current version includes a total of 83,422,341 nsSNVs and splice site SNVs (ssSNVs). It compiles prediction scores from 17 prediction algorithms. In addition, each file was analyzed by the companion dbscSNV database, which includes all potential human SNV within splicing consensus regions (−3 to +8 at the 5′ splice site and −12 to +2 at the 3′ splice site), i.e. splicing consensus regions (scSNVs), and predictions for their potential of altering splicing.

Quality control and prioritization

Pooled sequencing, the initial quality analysis allow only variants with the quality read depth of bases with Phred score ≥30 and average per-pool sample depth of bases with Phred score ≥30 were subjected for further analysis. Low variant counts per pool (<12) were also filtered out. All variants were then additionally filtered using a Forward/Reverse stand balance between 10-90% (strand bias). Because of the study focus on the low frequency variants (MAF <5%) all variants with MAF ≥5% were removed from further analysis.

Verification of selected variants by individual genotyping

Individual genotyping for selected markers in individual DNA samples was performed using a custom Sequenom iPLEX assay in conjunction with the Mass ARRAY platform (Sequenom Inc., La Jolla, CA, USA) Panels of SNP markers were designed using

Sequenom Assay Design 3.2 software (Sequenom Inc., La Jolla, CA, USA), similar to previously described methodology from our laboratory [9, 16, 17].

<u>Statistic tests and calculations</u>

CMAT is a pooling method proposed by Zawistowski et al., that works by comparing minor allele counts (for cases and controls) against the major-allele counts (for cases and controls) [18]. The calculation of CMAT were performed using AssotesR package (0.1-10) from CRAN repository (<u>cran.r-project.org/package=AssotesteR</u>) and written by Gaston Sanchez (gastonsanchez.com/) as documented at <u>www.rdocumentation.org/packages/AssotesteR/versions/0.1-10</u>. CMAT test was performed only for genes or region with at least one allele containing damaging variants in either investigated group. If no damaging variants were observed in one of the cohorts (controls or ischemic stroke), the exact Fisher test was used for the statistical analysis of the difference in frequency of damaging variants between study groups.

2. **<u>Fluorescence-based assay for P2Y1 and P2Y12 receptor activation in L cells</u>**

   Plasmid constructs: Wild-type P2RY1, P2RY12 and GIRK4 S143T cDNA constructs (in pcDNA3.1) were kind gifts from Dr. Henry L. Puhl, Ph.D. (NIH/NIAAA). The P2RY1 C824A, P2RY1 C755A, P2RY12 G672T and P2RY12 C550A mutant constructs were prepared by Watsonbio (Houston, TX). The fidelity of the mutations for each variant was confirmed by Sanger sequencing.

   Cell culture: For this set of experiments, we employed the mouse fibroblast line, L cells (ATCC). The cells were maintained in Dulbecco's modified Eagle's medium (DMEM) supplemented with 10% fetal bovine serum, 1% penicillin/streptomycin/glutamine and 10% non-essential amino acids.

Membrane potential assay: The FlexStation 3 Plate Reader (Molecular Devices) was employed to measure the fluorescence changes. The blue FLIPR membrane potential dye (Molecular Devices) was reconstituted in non-supplemented DMEM (without phenol red). Thereafter, the DMEM was aspirated from the wells and the cells were loaded with 90 l of dye solution and 90 l of DMEM (without phenol red) for at least 30 min in the cell incubator. After the cells were loaded, the plate

with the cells and plate containing MeSADP (1, 0.3 and 0.1 M) were inserted into the plate reader. Data acquisition was performed with SoftMax Pro software (Molecular Devices) in which the dye was excited at a wavelength of 530 nm and emitted at 565 nm. Fluorescence readings were acquired every 1.6 sec and MeSADP was applied to each well 20 sec after the start of the recording. The data obtained was performed in triplicate. The raw fluorescence data is plotted as the change in relative fluorescence units (RFU) before and after MeSADP application. The summary data is presented as mean (± SEM) % change in RFU for each concentration employed. Data and statistical analysis were performed with Prism (GraphPad software) with a p value < 0.05 considered statistically significant. The graphs and fluorescence traces were generated with Autodesk Graphic.

Table S1. Full list of all (common and low frequency with rare variants) known variants (dbSNP138) observed during re-sequencing of 26 genes in the study, including synonymous, nonsynonymous, non-coding and intergenic variants.

Chr	Position	Ref	Alt	Locus	Gene	dbSNP138	Function
chr3	4856234	G	A	exonic	*ITPR1*	rs901854	synonymous
chr19	55539072	T	G	exonic	*GP6*	rs892090	synonymous
chr1	156882996	G	C	exonic	*PEAR1*	rs822441	synonymous
chr1	156877797	C	A	exonic	*PEAR1*	rs77235035	synonymous
chr3	4767262	T	C	exonic	*ITPR1*	rs7613447	synonymous
chr11	63987913	G	A	exonic	*FERMT3*	rs72920390	synonymous
chr3	4856180	T	C	exonic	*ITPR1*	rs711631	synonymous
chr3	152554357	A	G	exonic	*P2RY1*	rs701265	synonymous
chr3	151056598	A	C	exonic	*P2RY12*	rs6809699	synonymous
chr3	151056616	G	A	exonic	*P2RY12*	rs6785930	synonymous
chr1	156878737	T	C	exonic	*PEAR1*	rs6671392	synonymous
chr3	4817057	T	C	exonic	*ITPR1*	rs6442905	synonymous
chr13	47469940	G	A	exonic	*HTR2A*	rs6313	synonymous
chr13	47466622	G	A	exonic	*HTR2A*	rs6305	synonymous
chr3	4774816	C	T	exonic	*ITPR1*	rs61757111	synonymous
chr5	52347366	A	C	exonic	*ITGA2*	rs61737774	synonymous
chr1	169566326	G	A	exonic	*SELP*	rs6135	synonymous
chr1	169566308	G	A	exonic	*SELP*	rs6132	synonymous
chr1	169586330	C	T	exonic	*SELP*	rs6129	synonymous
chr1	169562904	C	T	exonic	*SELP*	rs6128	synonymous
chr3	128780714	G	A	exonic	*GP9*	rs6069	synonymous
chr17	4836973	A	G	exonic	*GP1BA*	rs6067	synonymous
chr17	4836673	C	T	exonic	*GP1BA*	rs6066	synonymous
chr7	80292449	G	A	exonic	*CD36*	rs5956	synonymous
chr17	45364540	T	C	exonic	*ITGB3*	rs5919	synonymous
chr17	42452054	C	T	exonic	*ITGA2B*	rs5913	synonymous
chr17	42457120	C	T	exonic	*ITGA2B*	rs5912	synonymous

chr17	42449789	G	A	exonic	*ITGA2B*	rs5910	synonymous
chr19	3600198	C	T	exonic	*TBXA2R*	rs5748	synonymous
chr19	3600390	G	A	exonic	*TBXA2R*	rs5745	synonymous
chr1	156884584	C	T	exonic	*PEAR1*	rs56260937	synonymous
chr1	156883242	G	A	exonic	*PEAR1*	rs55864969	synonymous
chr12	6954875	C	T	exonic	*GNB3*	rs5443	synonymous
chr19	55539049	C	T	exonic	*GP6*	rs5030705	synonymous
chr17	45369777	A	G	exonic	*ITGB3*	rs4642	synonymous
chr17	45369789	G	A	exonic	*ITGB3*	rs4634	synonymous
chr12	6953100	T	A	exonic	*GNB3*	rs45476395	synonymous
chr19	3595794	A	G	exonic	*TBXA2R*	rs4523	synonymous
chr17	4837243	C	T	exonic	*GP1BA*	rs41466145	synonymous
chr3	4714841	G	A	exonic	*ITPR1*	rs41289636	synonymous
chr11	63988102	C	T	exonic	*FERMT3*	rs3802933	synonymous
chr11	63988045	G	A	exonic	*FERMT3*	rs3802932	synonymous
chr17	42463481	G	A	exonic	*ITGA2B*	rs375882355	synonymous
chr1	156879580	C	T	exonic	*PEAR1*	rs3737224	synonymous
chr19	17000676	C	G	exonic	*F2RL3*	rs370953951	synonymous
chr5	52369086	G	A	exonic	*ITGA2*	rs3213805	synonymous
chr5	52368472	C	T	exonic	*ITGA2*	rs3212583	synonymous
chr5	52351876	A	G	exonic	*ITGA2*	rs3212523	synonymous
chr5	52351437	C	T	exonic	*ITGA2*	rs3212521	synonymous
chr5	52369002	G	A	exonic	*ITGA2*	rs3212327	synonymous
chr3	4716811	A	C	exonic	*ITPR1*	rs2306877	synonymous
chr3	4712413	G	A	exonic	*ITPR1*	rs2306875	synonymous
chr3	4699936	G	C	exonic	*ITPR1*	rs2306869	synonymous
chr3	4842231	C	T	exonic	*ITPR1*	rs2291862	synonymous
chr19	47124714	T	G	exonic	*PTGIR*	rs2229129	synonymous
chr19	47127324	C	G	exonic	*PTGIR*	rs2229128	synonymous
chr3	194118946	C	T	exonic	*GP5*	rs202032002	synonymous
chr5	76029319	G	A	exonic	*F2R*	rs200544128	synonymous
chr3	4836852	G	A	exonic	*ITPR1*	rs200426774	synonymous
chr3	4726848	G	A	exonic	*ITPR1*	rs199960483	synonymous
chr10	112838892	C	A	exonic	*ADRA2A*	rs1800038	synonymous
chr11	63979162	C	T	exonic	*FERMT3*	rs17851033	synonymous
chr11	72946020	C	T	exonic	*P2RY2*	rs1783596	synonymous
chr11	72946308	T	A	exonic	*P2RY2*	rs17244555	synonymous
chr19	55525894	G	A	exonic	*GP6*	rs1671151	synonymous
chr19	55538980	T	C	exonic	*GP6*	rs1654425	synonymous
chr17	45368337	A	C	exonic	*ITGB3*	rs15908	synonymous
chr5	52353922	T	A	exonic	*ITGA2*	rs149911770	synonymous
chr3	194118763	G	A	exonic	*GP5*	rs149317860	synonymous
chr1	169586363	G	A	exonic	*SELP*	rs147922476	synonymous
chr11	72945513	G	T	exonic	*P2RY2*	rs144543190	synonymous
chr5	76028467	G	A	exonic	*F2R*	rs143038729	synonymous
chr22	19711765	C	T	exonic	*GP1BB*	rs142352780	synonymous
chr11	63990865	C	T	exonic	*FERMT3*	rs142025489	synonymous
chr1	156883194	G	A	exonic	*PEAR1*	rs141857901	synonymous
chr7	80292467	A	T	exonic	*CD36*	rs141680676	synonymous

chr13	47409005	G	A	exonic	*HTR2A*	rs139888059	synonymous
chr11	72945666	C	A	exonic	*P2RY2*	rs139591958	synonymous
chr1	169578860	C	T	exonic	*SELP*	rs138017338	synonymous
chr3	4829732	A	G	exonic	*ITPR1*	rs13079522	synonymous
chr1	156873727	G	A	exonic	*PEAR1*	rs12407843	synonymous
chr3	194118874	T	C	exonic	*GP5*	rs1223989	synonymous
chr3	4776960	C	T	exonic	*ITPR1*	rs113368815	synonymous
chr19	3595923	G	A	exonic	*TBXA2R*	rs1131882	synonymous
chr3	4558241	G	A	exonic	*ITPR1*	rs112944532	synonymous
chr5	52347369	C	T	exonic	*ITGA2*	rs1126643	synonymous
chr1	156878531	T	C	exonic	*PEAR1*	rs11264580	synonymous
chr3	152553628	C	T	exonic	*P2RY1*	rs1065776	synonymous
chr1	169581608	G	A	exonic	*SELP*	rs139249907	stopgain
chr1	156883215	C	A	exonic	*PEAR1*	rs822442	nonsynonymous
chr19	17000632	G	A	exonic	*F2RL3*	rs773902	nonsynonymous
chr17	42463054	G	C	exonic	*ITGA2B*	rs76066357	nonsynonymous
chr17	42453084	C	T	exonic	*ITGA2B*	rs74988902	nonsynonymous
chr11	72946279	T	C	exonic	*P2RY2*	rs74472890	nonsynonymous
chr13	47409034	G	A	exonic	*HTR2A*	rs6314	nonsynonymous
chr13	47470824	C	T	exonic	*HTR2A*	rs6312	nonsynonymous
chr13	47409048	G	A	exonic	*HTR2A*	rs6308	nonsynonymous
chr1	169580885	C	T	exonic	*SELP*	rs6131	nonsynonymous
chr1	169566313	C	T	exonic	*SELP*	rs6127	nonsynonymous
chr1	169582317	C	T	exonic	*SELP*	rs6125	nonsynonymous
chr17	4836381	C	T	exonic	*GP1BA*	rs6065	nonsynonymous
chr17	45360730	T	C	exonic	*ITGB3*	rs5918	nonsynonymous
chr17	42453065	A	C	exonic	*ITGA2B*	rs5911	nonsynonymous
chr17	45363765	A	G	exonic	*ITGB3*	rs56173532	nonsynonymous
chr5	52344487	A	G	exonic	*ITGA2*	rs55973669	nonsynonymous
chr3	12641707	C	T	exonic	*RAF1*	rs555034652	nonsynonymous
chr12	6954864	G	A	exonic	*GNB3*	rs5442	nonsynonymous
chr19	47126849	G	A	exonic	*PTGIR*	rs4987262	nonsynonymous
chr1	156882757	C	G	exonic	*PEAR1*	rs41299597	nonsynonymous
chr3	4704816	G	A	exonic	*ITPR1*	rs41289628	nonsynonymous
chr19	55525818	C	T	exonic	*GP6*	rs41275822	nonsynonymous
chr3	128781048	G	A	exonic	*GP9*	rs3796130	nonsynonymous
chr11	72946140	G	C	exonic	*P2RY2*	rs3741156	nonsynonymous
chr3	4821291	G	T	exonic	*ITPR1*	rs373973399	nonsynonymous
chr3	4714920	A	G	exonic	*ITPR1*	rs35789999	nonsynonymous
chr11	72945341	C	T	exonic	*P2RY2*	rs2511241	nonsynonymous
chr19	55527081	C	T	exonic	*GP6*	rs2304167	nonsynonymous
chr19	55526373	G	C	exonic	*GP6*	rs2304166	nonsynonymous
chr7	80293767	G	T	exonic	*CD36*	rs201715989	nonsynonymous
chr19	17001214	G	A	exonic	*F2RL3*	rs201593664	nonsynonymous
chr5	76029200	A	G	exonic	*F2R*	rs201571376	nonsynonymous
chr3	4716885	C	T	exonic	*ITPR1*	rs201519806	nonsynonymous
chr17	4837662	T	C	exonic	*GP1BA*	rs201408072	nonsynonymous
chr3	4842276	G	A	exonic	*ITPR1*	rs201144431	nonsynonymous
chr19	55525763	A	C	exonic	*GP6*	rs200566792	nonsynonymous

chr17	42455791	G	A	exonic	*ITGA2B*	rs200481952	nonsynonymous
chr19	47127439	A	G	exonic	*PTGIR*	rs200213497	nonsynonymous
chr19	3594967	G	A	exonic	*TBXA2R*	rs199908583	nonsynonymous
chr3	4725441	A	G	exonic	*ITPR1*	rs199698357	nonsynonymous
chr19	55543660	G	A	exonic	*GP6*	rs199588110	nonsynonymous
chr19	55526345	T	G	exonic	*GP6*	rs1671152	nonsynonymous
chr19	55530035	C	T	exonic	*GP6*	rs1654416	nonsynonymous
chr19	55526359	A	T	exonic	*GP6*	rs1654413	nonsynonymous
chr11	72946204	C	T	exonic	*P2RY2*	rs1626154	nonsynonymous
chr19	55536595	G	A	exonic	*GP6*	rs1613662	nonsynonymous
chr11	72945434	T	C	exonic	*P2RY2*	rs148391446	nonsynonymous
chr17	45376796	G	A	exonic	*ITGB3*	rs144884023	nonsynonymous
chr11	63974995	C	G	exonic	*FERMT3*	rs142815441	nonsynonymous
chr11	72945799	A	G	exonic	*P2RY2*	rs141776297	nonsynonymous
chr1	169572405	T	A	exonic	*SELP*	rs139642713	nonsynonymous
chr19	17000518	G	A	exonic	*F2RL3*	rs139190744	nonsynonymous
chr11	72946298	C	T	exonic	*P2RY2*	rs138929283	nonsynonymous
chr1	28477192	T	C	exonic	*PTAFR*	rs138629813	nonsynonymous
chr1	156883546	A	G	exonic	*PEAR1*	rs12137505	nonsynonymous
chr1	156883493	G	A	exonic	*PEAR1*	rs11264581	nonsynonymous
chr17	45360680	C	T	intronic	*ITGB3*	rs988684	
chr3	4859725	G	A	intronic	*ITPR1*	rs9844268	
chr17	42454270	T	C	intronic	*ITGA2B*	rs850731	
chr17	42454463	G	C	intronic	*ITGA2B*	rs850730	
chr17	4835852	A	T	intronic	*GP1BA*	rs81663	
chr3	4704907	G	A	intronic	*ITPR1*	rs80123990	
chr3	4821374	G	A	intronic	*ITPR1*	rs78956048	
chr11	63990505	C	G	intronic	*FERMT3*	rs78324705	
chr19	16999897	T	C	UTR5	*F2RL3*	rs773905	
chr19	17000131	G	A	intronic	*F2RL3*	rs773904	
chr19	17000231	C	T	intronic	*F2RL3*	rs773903	
chr3	4856285	G	A	intronic	*ITPR1*	rs76604555	
chr3	4703937	C	T	intronic	*ITPR1*	rs7632000	
chr3	4708997	G	T	intronic	*ITPR1*	rs7625003	
chr5	52285453	G	A	intronic	*ITGA2*	rs75823056	
chr1	156884365	T	C	intronic	*PEAR1*	rs749256	
chr20	44747086	G	A	intronic	*CD40*	rs745307	
chr1	169564167	G	C	intronic	*SELP*	rs742127	
chr1	156876729	T	C	intronic	*PEAR1*	rs735953	
chr3	4726947	T	G	intronic	*ITPR1*	rs733018	
chr1	169599254	T	C	intronic	*SELP*	rs732314	
chr17	42454342	A	G	intronic	*ITGA2B*	rs71371995	
chr3	4703657	T	A	intronic	*ITPR1*	rs6801737	
chr1	156876221	G	A	intronic	*PEAR1*	rs6688349	
chr1	156876206	G	A	intronic	*PEAR1*	rs6688345	
chr1	156875037	T	G	intronic	*PEAR1*	rs6676171	
chr1	156876237	C	T	intronic	*PEAR1*	rs6664765	
chr3	4856993	C	G	intronic	*ITPR1*	rs6442911	
chr3	4694010	A	C	intronic	*ITPR1*	rs6442896	

chr3	4693937	G	C	intronic	*ITPR1*	rs6442895	
chr1	156883617	G	A	intronic	*PEAR1*	rs61813833	
chr1	156882950	C	T	intronic	*PEAR1*	rs61813832	
chr20	44751040	A	G	intronic	*CD40*	rs61760051	
chr19	55539308	C	G	intronic	*GP6*	rs61145631	
chr19	55539303	C	G	intronic	*GP6*	rs59293899	
chr1	156882261	C	T	intronic	*PEAR1*	rs57731889	
chr3	12641425	T	C	intronic	*RAF1*	rs5746223	
chr1	156875016	A	T	intronic	*PEAR1*	rs574339307	
chr3	4694011	G	A	intronic	*ITPR1*	rs56896093	
chr19	3595981	G	A	intronic	*TBXA2R*	rs56321318	
chr17	42449695	G	A	UTR3	*ITGA2B*	rs56311858	
chr17	45331397	G	A	intronic	*ITGB3*	rs56221506	
chr7	80302798	G	T	intronic	*CD36*	rs56082629	
chr17	42462844	G	T	intronic	*ITGA2B*	rs544596241	
chr5	52374512	T	G	intronic	*ITGA2*	rs540079113	
chr11	63988675	C	T	intronic	*FERMT3*	rs537670548	
chr17	42455650	C	A	intronic	*ITGA2B*	rs527980644	
chr3	4722956	A	G	intronic	*ITPR1*	rs4684438	
chr1	156875097	G	A	intronic	*PEAR1*	rs4661075	
chr1	156875092	G	A	intronic	*PEAR1*	rs4661074	
chr17	62406986	G	A	intronic	*PECAM1*	rs45495798	
chr17	45368248	G	A	intronic	*ITGB3*	rs41315064	
chr3	4716708	C	T	intronic	*ITPR1*	rs41311607	
chr3	4808477	G	A	intronic	*ITPR1*	rs41308265	
chr3	4695661	C	T	intronic	*ITPR1*	rs41308220	
chr3	4703688	T	A	intronic	*ITPR1*	rs41305884	
chr3	4704668	G	A	intronic	*ITPR1*	rs41289626	
chr3	4687498	T	A	intronic	*ITPR1*	rs41289624	
chr20	44755376	G	C	intronic	*CD40*	rs41282788	
chr19	55543973	C	T	intronic	*GP6*	rs41275824	
chr1	156881959	C	T	intronic	*PEAR1*	rs41273215	
chr1	156879504	A	G	intronic	*PEAR1*	rs41273205	
chr1	156874421	C	G	intronic	*PEAR1*	rs41267433	
chr1	169559326	G	A	intronic	*SELP*	rs3917853	
chr1	169580717	T	C	intronic	*SELP*	rs3917731	
chr1	169582401	C	G	intronic	*SELP*	rs3917721	
chr1	169588354	G	A	intronic	*SELP*	rs3917696	
chr5	52369193	A	G	intronic	*ITGA2*	rs3815755	
chr7	80302196	G	A	intronic	*CD36*	rs3807075	
chr17	42463176	G	A	intronic	*ITGA2B*	rs377288289	
chr20	44757407	G	A	intronic	*CD40*	rs3765459	
chr20	44757213	A	G	intronic	*CD40*	rs3765457	
chr17	45380002	T	C	intronic	*ITGB3*	rs3760372	
chr11	63979074	C	T	intronic	*FERMT3*	rs376021574	
chr3	4738774	A	G	intronic	*ITPR1*	rs3749382	
chr3	4768756	A	G	intronic	*ITPR1*	rs373579234	
chr17	42455953	G	A	intronic	*ITGA2B*	rs370496752	
chr5	52369122	A	G	intronic	*ITGA2*	rs369350934	

chr3	12641345	T	C	intronic	*RAF1*	rs369196677	
chr5	52382703	T	C	intronic	*ITGA2*	rs3212698	
chr5	52382748	A	T	intronic	*ITGA2*	rs3212635	
chr5	52370174	G	A	intronic	*ITGA2*	rs3212591	
chr5	52368594	C	T	intronic	*ITGA2*	rs3212586	
chr5	52368546	T	C	intronic	*ITGA2*	rs3212584	
chr5	52368366	A	C	intronic	*ITGA2*	rs3212582	
chr5	52367715	T	C	intronic	*ITGA2*	rs3212580	
chr5	52356692	T	C	intronic	*ITGA2*	rs3212538	
chr5	52353959	T	C	intronic	*ITGA2*	rs3212529	
chr5	52351746	G	A	intronic	*ITGA2*	rs3212522	
chr5	52337908	C	T	intronic	*ITGA2*	rs3212441	
chr7	80301218	C	G	intronic	*CD36*	rs3212017	
chr7	80295701	T	C	intronic	*CD36*	rs3212012	
chr7	80301369	C	A	intronic	*CD36*	rs3211942	
chr7	80293916	C	T	intronic	*CD36*	rs3211908	
chr7	80290369	A	G	intronic	*CD36*	rs3211892	
chr3	4562910	G	C	intronic	*ITPR1*	rs304015	
chr3	4693943	G	A	intronic	*ITPR1*	rs28724276	
chr3	4693953	G	A	intronic	*ITPR1*	rs28592010	
chr3	4693977	G	A	intronic	*ITPR1*	rs28562557	
chr12	6950403	G	A	intronic	*GNB3*	rs28395781	
chr17	62401118	T	C	UTR3	*PECAM1*	rs2812	
chr5	52322721	G	T	intronic	*ITGA2*	rs26678	
chr1	156875107	C	A	intronic	*PEAR1*	rs2644590	
chr3	12645007	C	T	intronic	*RAF1*	rs2596828	
chr3	12650482	T	A	intronic	*RAF1*	rs2454440	
chr3	4669692	G	A	intronic	*ITPR1*	rs2307067	
chr3	4725239	G	A	intronic	*ITPR1*	rs2306878	
chr3	4712710	A	T	intronic	*ITPR1*	rs2306876	
chr3	4699727	T	C	intronic	*ITPR1*	rs2306871	
chr3	4699776	T	C	intronic	*ITPR1*	rs2306870	
chr3	4699967	C	G	intronic	*ITPR1*	rs2306868	
chr3	4856650	T	C	intronic	*ITPR1*	rs2304820	
chr5	52355854	T	C	intronic	*ITGA2*	rs2303127	
chr5	52366162	G	A	intronic	*ITGA2*	rs2303126	
chr13	47466781	T	G	intronic	*HTR2A*	rs2296973	
chr17	45367681	C	T	intronic	*ITGB3*	rs2292864	
chr3	12633168	A	G	intronic	*RAF1*	rs2290161	
chr19	55543834	C	T	intronic	*GP6*	rs2288905	
chr5	52367706	T	C	intronic	*ITGA2*	rs2287871	
chr3	12641518	A	G	intronic	*RAF1*	rs2246390	
chr17	4835895	T	C	UTR5	*GP1BA*	rs2243093	
chr5	76029372	T	C	UTR3	*F2R*	rs2227800	
chr5	52366138	G	A	intronic	*ITGA2*	rs2112290	
chr17	62406971	A	G	intronic	*PECAM1*	rs2070783	
chr19	55527233	C	T	intronic	*GP6*	rs2019599	
chr3	4741447	G	A	intronic	*ITPR1*	rs201483668	
chr1	156882188	C	G	intronic	*PEAR1*	rs201049430	

chr17	42452338	G	A	intronic	*ITGA2B*	rs200677976	
chr1	156884391	G	C	intronic	*PEAR1*	rs200494787	
chr7	80299255	C	T	intronic	*CD36*	rs200439592	
chr5	52285405	G	A	intronic	*ITGA2*	rs200410974	
chr12	6950361	C	T	intronic	*GNB3*	rs189513536	
chr11	63978072	G	A	intronic	*FERMT3*	rs188768294	
chr17	45376921	C	T	intronic	*ITGB3*	rs188471209	
chr20	44746982	T	C	UTR5	*CD40*	rs1883832	
chr20	44750444	C	T	intronic	*CD40*	rs187683423	
chr3	12626792	A	G	intronic	*RAF1*	rs187514758	
chr5	76012370	C	G	intronic	*F2R*	rs186204177	
chr17	4835872	C	T	intronic	*GP1BA*	rs183989314	
chr3	4829575	T	C	intronic	*ITPR1*	rs17786144	
chr3	4725555	G	C	intronic	*ITPR1*	rs17710726	
chr1	169563062	G	A	intronic	*SELP*	rs17522707	
chr17	45367244	G	C	intronic	*ITGB3*	rs17218711	
chr17	45367669	G	A	intronic	*ITGB3*	rs16941829	
chr19	55529933	A	G	intronic	*GP6*	rs1671192	
chr19	55527189	G	T	intronic	*GP6*	rs1671153	
chr1	169564130	T	G	intronic	*SELP*	rs1569471	
chr1	156879929	G	A	intronic	*PEAR1*	rs149157097	
chr3	4767199	G	C	intronic	*ITPR1*	rs147708579	
chr1	156880288	C	G	intronic	*PEAR1*	rs146834392	
chr17	42452153	G	A	intronic	*ITGA2B*	rs145564830	
chr13	47466439	G	A	intronic	*HTR2A*	rs145194929	
chr5	52285286	G	A	UTR5	*ITGA2*	rs143667535	
chr11	63987559	G	A	intronic	*FERMT3*	rs143587409	
chr5	52344610	A	G	intronic	*ITGA2*	rs1421933	
chr17	4837906	A	G	UTR3	*GP1BA*	rs142179565	
chr1	169588501	C	T	intronic	*SELP*	rs142170818	
chr17	62406985	C	T	intronic	*PECAM1*	rs141531322	
chr1	169582130	T	G	intronic	*SELP*	rs141161996	
chr5	52285389	G	A	intronic	*ITGA2*	rs139897689	
chr5	52360658	A	T	intronic	*ITGA2*	rs139704993	
chr11	63987641	A	T	intronic	*FERMT3*	rs138055838	
chr5	52338083	T	G	intronic	*ITGA2*	rs1363192	
chr1	169560624	G	A	intronic	*SELP*	rs13306837	
chr17	45364619	A	G	intronic	*ITGB3*	rs13306478	
chr5	52386458	G	A	UTR3	*ITGA2*	rs13173706	
chr3	4716672	T	C	intronic	*ITPR1*	rs13082052	
chr17	42455717	G	A	intronic	*ITGA2B*	rs12938868	
chr19	47126614	C	G	intronic	*PTGIR*	rs12459883	
chr1	156874993	C	T	intronic	*PEAR1*	rs12048392	
chr3	4776784	A	G	intronic	*ITPR1*	rs11920001	
chr17	45331358	C	G	intronic	*ITGB3*	rs11871407	
chr17	45377825	T	C	intronic	*ITGB3*	rs11870252	
chr17	45378041	C	T	intronic	*ITGB3*	rs11867253	
chr1	156878887	G	A	intronic	*PEAR1*	rs11810027	
chr5	52351995	T	A	intronic	*ITGA2*	rs118050125	

chr1	156878435	C	T	intronic	*PEAR1*	rs11800463	
chr20	44751415	A	G	intronic	*CD40*	rs11699100	
chr20	44757475	C	T	intronic	*CD40*	rs11697349	
chr19	3594784	C	T	UTR3	*TBXA2R*	rs116937991	
chr1	156884399	G	C	intronic	*PEAR1*	rs114364739	
chr5	52386464	A	C	UTR3	*ITGA2*	rs113222066	
chr19	55543448	G	A	intronic	*GP6*	rs111888556	
chr3	4562667	T	G	intronic	*ITPR1*	rs1038639	
chr17	45377712	C	T	intronic	*ITGB3*	rs10221263	

Table S2. List of all indels (common and rare) observed in 26 sequenced genes in patients from both cohorts (ischemic stroke and controls) investigated in the study.

Chr	Start	End	Ref	Alt	dbSNP 138	Function	Gene	ExonicFunc	AAChange
chr3	4669376	4669376	T	-		intronic	*ITPR1*		
chr3	4687470	4687470	-	CT	rs5846330	intronic	*ITPR1*		
chr3	4702858	4702858	-	T	rs11446330	intronic	*ITPR1*		
chr3	4738999	4738999	-	GTGT	rs35795762	intronic	*ITPR1*		
chr17	4837499	4837500	TA	-	.	exonic	*GP1BA*	frameshift deletion	c.1600_1601del:p.Y534fs
chr17	4837744	4837748	TAATG	-	.	exonic	*GP1BA*	frameshift deletion	c.1845_1849del:p.P615fs
chr17	4837744	4837748	TAATG	-	.	exonic	*GP1BA*	frameshift deletion	c.1845_1849del:p.P615fs
chr17	4837952	4837952	-	G	rs199616811	UTR3	*GP1BA*		
chr3	4878420	4878420	T/TTTTTTTTT	-	rs201029025	intronic	*ITPR1*		
chr12	6952414	6952416	CCT	-		intronic	*GNB3*		
chr12	6955853	6955854	AC	-		intronic	*GNB3*		
chr12	6955865	6955865	-	CACA	rs199501562	intronic	*GNB3*		
chr17	42454567	42454567	T	-	rs376710190	intronic	*ITGA2B*		
chr17	45364391	45364395	TTTGT	-	rs56197296	intronic	*ITGB3*		
chr17	45377702	45377702	-	GCGT	rs60065475	intronic	*ITGB3*		
chr17	45377712	45377712	-	GT	rs58012849	intronic	*ITGB3*		
chr19	47127512	47127512	-	G	rs552005985	intronic	*PTGIR*		
chr13	47408902	47408902	-	T	rs58145637	UTR3	*HTR2A*		
chr5	52358762	52358762	A	-	.	intronic	*ITGA2*		
chr5	52365923	52365923	-	TT	rs398108915	intronic	*ITGA2*		
chr5	52370823	52370823	T	-		intronic	*ITGA2*		
chr5	52374528	52374531	TAAA	-	rs3212605	intronic	*ITGA2*		
chr19	55526103	55526103	-	CAGA	rs59110861	exonic	*GP6*	frameshift insertion	c.1209_1210insTCTG:p.P404fs
chr19	55527173	55527174	AC	-		intronic	*GP6*		
chr11	63990997	63990997	C	-	rs5792315	UTR3	*FERMT3*		
chr1	156874875	156874875	G	-	rs539280179	intronic	*PEAR1*		
chr1	156874875	156874876	GG	-	rs78436112	intronic	*PEAR1*		
chr1	156877884	156877884	-	G	rs150532135	intronic	*PEAR1*		
chr1	156881961	156881961	-	C	rs528242119	intronic	*PEAR1*		
chr1	169559334	169559334	T	-	rs3917852	intronic	*SELP*		

chr1	169560727	169560727	-	A	rs397942211	intronic	*SELP*		
chr1	169580892	169580892	-	TT	.	exonic	*SELP*	frameshift insertion	c.984_985insAA:p.A329fs
chr1	169580972	169580972	-	AT	rs61535831	intronic	*SELP*		
chr1	169586221	169586221	T	-	.	intronic	*SELP*		

Table S3. Demographic and clinical characteristics of the study population.

	**Ischemic stroke**	**Control**	P
**Female**	40%	41%	0.98
**Age**	70.5±12	70,00±11	0.99
**Hypertension**	77.40%	81.00%	0.07
**Heart failure**	16.60%	41.60%	<0.001*
**CAD**	29.40%	53.80%	<0.001*
**MI**	16.00%	34.60%	<0.001*
**Diabetes**	19.60%	56.60%	<0.001*
**Smokers**	38.4%	37%	0.69

CAD-Coronary artery disease, MI- Myocardial infarction

* statistically significant differences by Chi2 test or t test (for age differences)
